# Cytotoxicity induced by nanobacteria and nanohydroxyapatites in human choriocarcinoma cells

**DOI:** 10.1186/1556-276X-9-616

**Published:** 2014-11-14

**Authors:** Mingjun Zhang, Jinmei Yang, Jing Shu, Changhong Fu, Shengnan Liu, Ge Xu, Dechun Zhang

**Affiliations:** 1 Molecular Medicine and Tumor Research Center, Chongqing Medical University, Medical College Road, Yuzhong District, Chongqing 400016, People’s Republic of China; 2 Electron Microscopy Group, Department of Life Science, Chongqing Medical University, Chongqing, People’s Republic of China; 3 The First People’s Hospital of Jiulongpo District, Chongqing, People’s Republic of China

**Keywords:** Nanobacteria (NB), Nanohydroxyapatites (nHAPs), Human choriocarcinoma (JAR) cells, Cytotoxicity, Apoptosis, Autophagy

## Abstract

We explored the cytotoxic effects of nanobacteria (NB) and nanohydroxyapatites (nHAPs) against human choriocarcinoma cells (JAR) and the mechanisms of action underlying their cytotoxicity. JAR cells were co-cultured with NB and nHAPs for 48 h, and ultrastructural changes were more readily induced by NB than nHAPs. Autophagy in the plasma of JAR cells were observed in the NB group. The rate of apoptosis induced by NB was higher than that for nHAPs. The expression of Bax and FasR proteins in the NB group was stronger than that for the nHAP group. NB probably resulted in autophagic formation. Apoptosis was possibly activated via FasL binding to the FasR signaling pathway.

## Background

There is some debate as to whether nanobacteria (NB) are the smallest life forms on earth [1,2] or inorganic calcium-protein complexes [3-5]. However, in the past 20 years, NB is closely related to many calcification diseases, such as notably cholecystolithiasis, renal lithiasis, atherosclerosis, dental calculus, and so on [6-9]. In previous studies, we not only successfully isolated NB from human placental tissues and determined their 16S rRNA sequences ([GenBank:JF823648]) [10], but also found that the positive rate of nanobacteria in the calcified placenta and umbilical cord blood was significantly higher than that of the normal placenta [11]. These showed that NB was closely related to the placenta calcification. We speculated that nanobacteria may induce trophoblastic cell cytotoxicity, which resulted in the placenta calcification. Moreover, the self-replicating capacity of NB was shown by Lu et al. [12]. The cytotoxic effects between NB and nanohydroxyapatites (nHAPs) against human breast cancer cells were compared. The cytotoxic effects induced by NB were stronger than those induced by nHAPs [13]. Hunter et al. reported the formation of a NB biofilm during cultivation [14]. In the current study, we investigated the cytotoxicity induced by NB and nHAPs in human choriocarcinoma (JAR) cells and attempted to probe the relationship between placenta calcification and NB.

## Methods

### Cell culture

JAR cells (HTB-26™) were purchased from the American Type Culture Collection (ATCC, Manassas, VA, USA). Cells were cultured in DMEM-F12 (Hyclone, GE Healthcare Life Sciences, Logan, Utah) supplemented with penicillin (110 U/mL), streptomycin (100 μg/mL), 2 mM glutamine, and 10% fetal bovine serum (FBS; Gibco, Life Technologies, Carlsbad, CA, USA). Cultures were incubated at 37°C/5% CO_2_.

### NB cultures

The NB were isolated from the calcified placental tissue (which were derived from caesarean pregnant women at the First People's Hospital of Jiulongpo District in Chongqing) and cultured in 1640 RPMI with 10% FBS at 37°C/5% CO_2_. The culture media was refreshed every 3 days. The concentrations of NB were adjusted to 0.5, 1.0, and 2.0 meclary turbidity units (MCF) prior to measurement with a turbidity meter (LP2000-11, Shanghai Precise Instruments Corp., Shanghai, China).

### nHAP preparation

nHAP powder was purchased from Shangai Baoman Technical Corp. (Shanghai, China). nHAP solution was prepared by mixing 400 μg of powder with 2 mL of phosphate-buffered saline (PBS) and sonicated overnight. nHAP solutions at 0.5, 1.0, and 2.0 MCF were prepared by diluting with 1640 RPMI supplemented with 10% FBS, and their meclary turbidity was measured with a turbidity meter as NB.

### Hoechst 33258 staining

An apoptosis Hoechst 33258 fluorescence staining kit was provided by Beyotime Institute Biotechnology (Shanghai, China). We seeded 1 × 10^5^ JAR cells/well in 6-well plates containing coverslips overnight. Suspensions (200 μL/well) of NB (2 MCF), nHAP (2 MCF), or PBS (control) were added to wells when cells were 50% to 80% confluent. After 48 h, cells were collected and stained according to the manufacturer's protocol. Cells were observed using a fluorescence microscope and an excitation wavelength of 350 nm.

### Flow cytometry analysis

Apoptosis was assessed using flow cytometry (compuCyte) after the JAR cells were exposed to NB or nHAP suspensions for 48 h. We used the FITC-annexin V and propidium iodide (PI) apoptosis detection kit (Beyotime Institute Biotechnology) to detect the percentage of apoptotic and necrotic cells. The percentage of apoptotic and necrotic cells was calculated from the total cell numbers obtained by Cell Quest software. Double-negative cells were indicative of live cell populations. Annexin V-positive cells indicate early apoptotic cells. PI-positive cells indicate necrotic cells, and double-positive cells indicate late apoptotic or necrotic cells.

### Evaluation of cell morphology

JAR cells in 6-well plates were exposed to NB (2 MCF) or nHAP (2 MCF) for 48 h and were then observed by phase contrast microscopy (Olympus Corporation, Tokyo, Japan). Cultures were then fixed for 1 h with 2.5% glutaraldehyde and 1% osmic acid after washing three times with PBS. After dehydration through a graded series of cold ethanol, cells were embedded in epoxy resin. Ultrafine sections were stained with uranyl acetate and counterstained with lead citrate. The ultrastructure of JAR cells was examined using transmission electron microscopy (TEM; Hitachi-600, Tokyo, Japan).

### Western blotting

The Western Blot Kit, the Bax and Fas proteins, and supplementary reagents were purchased from Proteintech (Chicago, IL, USA). When the JAR cell cultures reached 50% to 80% confluence in 6-well plates, we added NB (2 MCF), nHAP (2 MCF), or PBS (control) to the wells (200 μL/well) for 48 h. The culture supernatant was aspirated and a lysis solution was added (100 μL/well) for 30 min at 4°C. Lysates were collected and subjected to Western blotting according to the manufacturer's protocols. Blots were visualized using BeyoECL Plus (BIO-RAD Molecular ImagerR ChemiDOC™XRS System, Bio*-*Rad Laboratories, Hercules, CA, USA).

### Ethics statement

The protocol was approved by the Ethics Committee of the Chongqing Medical University, Chongqing, China (Permit Number: 2011-2012003). Written informed consent was obtained from all the study subjects. All procedures followed the tenets of the Declaration of Helsinki.

### Statistical analysis

Data were analyzed using SPSS version 17 (SPSS Inc.) and PRISM 5 (GraphPad Software, Inc.).The *t*-test was used to analyze statistical significance between conditions, and one-way and two-way ANOVAs to analyze statistical results among several groups. A *p* value less than 0.05 was considered significant.

## Results

### TEM analysis of NB

**Figure 1:**
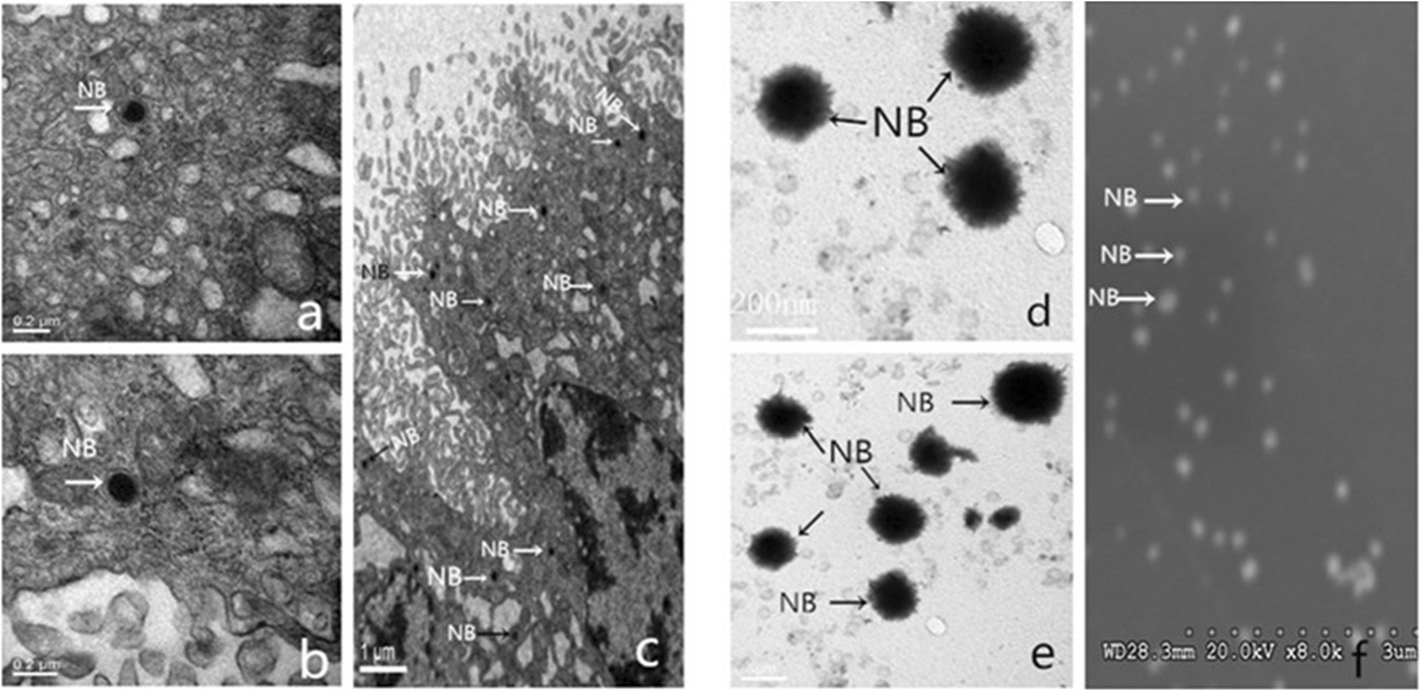
**Morphology of NB.** The morphology of NB under TEM and SEM: **(a-c)** in freshly fixed calcified placental tissues and **(d, e)** in the cultivated tissue under TEM. **(f)** Morphology of NB using scanning electron microscopy.

Fresh calcified human placental tissue was divided into two parts. The first part was cut into small pieces (about 1 mm^3^) and viewed using TEM. The second part was isolated and cultivated as described by Guo [10] and then subjected to TEM. The morphology of NB in freshly fixed calcified placental tissues (Figure 1a,b,c) was similar to that for the cultivated tissue (Figure 1d,e,f). The NB were typically coccoid and of different sizes ranging from 80 to 500 nm in diameter [15,16].

### Apoptosis

**Figure 2:**
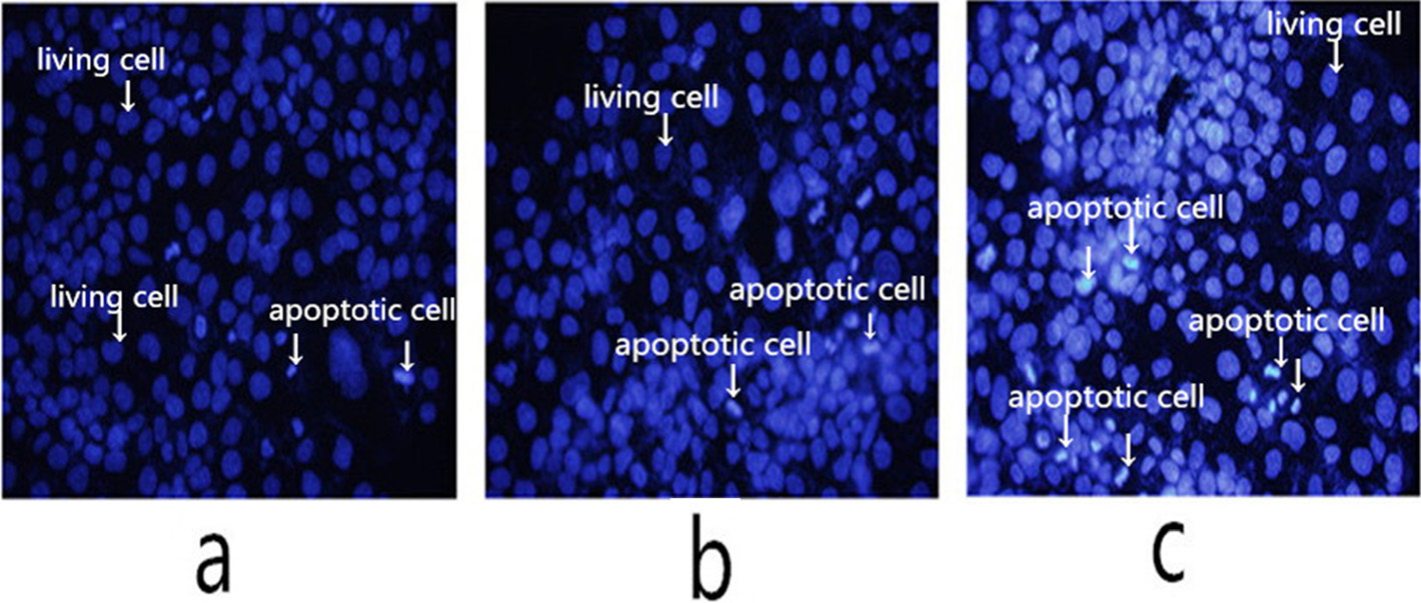
Stained nuclei of JAR cells following treatment with PBS (a), nHAPs (b), and NB (c) by fluorescence microscope (×400).

Using the fluorescence staining kit, the nuclei of alive cells were stained blue and the nuclei of apoptotic cells were stained white (Figure 2a,b,c). Apoptosis of JAR cells was significantly greater in the NB group compared with that in the nHAP group after a 48-h treatment.

### Flow cytometry analysis

**Figure 3:**
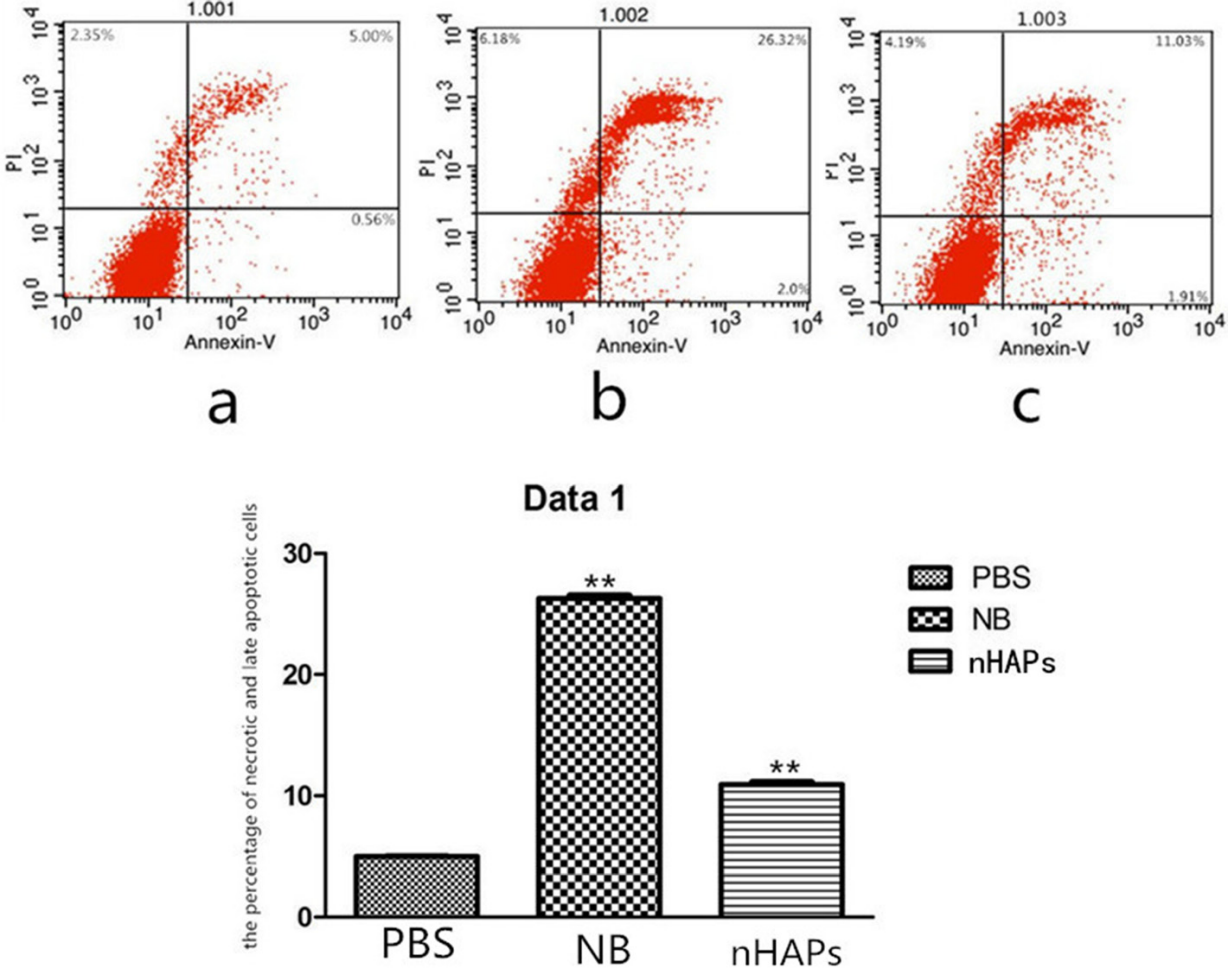
**Flow cytometry analysis.** Flow cytometry analysis of annexin V staining in cells following treatment with PBS **(a)**, NB **(b)**, or nHAPs **(c)**. **p <0.01.

The proportion of annexin V- and PI-positive cells was determined by flow cytometry (Figure 3a,b,c). Events in the lower right quadrants indicated the percentage of early apoptotic cells (annexin V-positive and PI-negative), while events in the upper right quadrants indicated the percentage of necrotic and late apoptotic cells (annexin V- and PI-positive). After the 48-h treatment, a significant difference was seen in the NB (26.3% ±1.2% vs. 5.0% ±0.8%) and nHAP (11.0% ±1.0% vs. 5.0% ±0.8%) groups compared with the control group (p <0.01 and p <0.05, respectively). The proportion of necrotic and late apoptotic cells in the NB group (26.3% ±1.2%) was significantly higher than that in the nHAP group (11.0% ±1.0%, *p* <0.01).

### Effects of nanoparticles on cell ultrastructure

**Figure 4:**
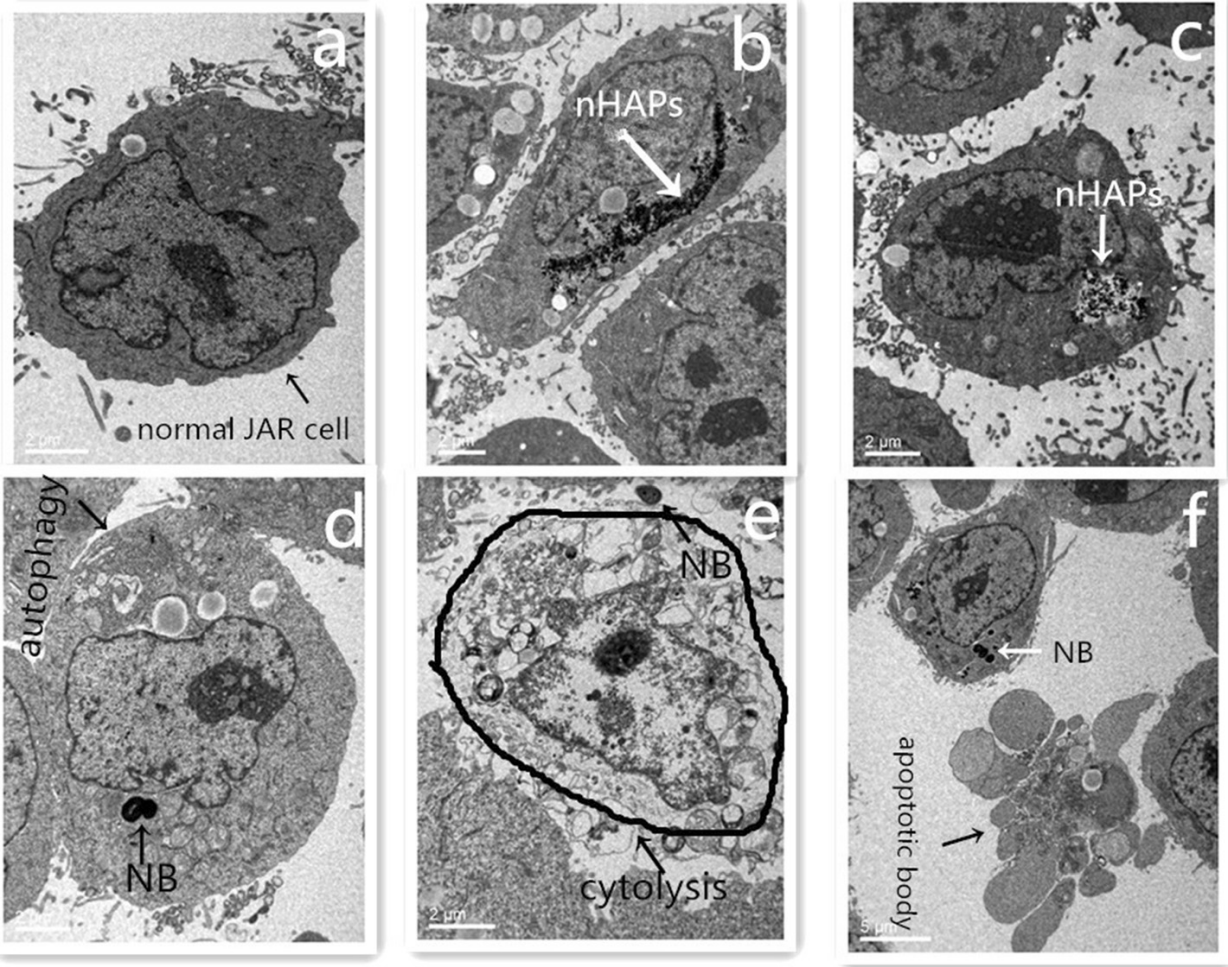
**TEM analysis showing the uptake of NB and nHAPs by JAR cells. (a)** Control group: JAR cells treated with PBS. **(b, c)** nHAP group: JAR cells treated with nHAPs. **(d-f)** NB group: JAR cells treated with NB.

Differences in cellular ultrastructure were observed in JAR cells treated with NB or nHAPs. TEM confirmed that nanoparticles were internalized into intracellular endosomes. Evidence of autophagy and cytolysis and the formation of apoptotic bodies were seen after 48 h of NB treatment (Figure 4d,e,f), but these changes were absent in the control group (Figure 4a) and in the nHAP group (Figure 4b,c).

### Western blotting

**Figure 5:**
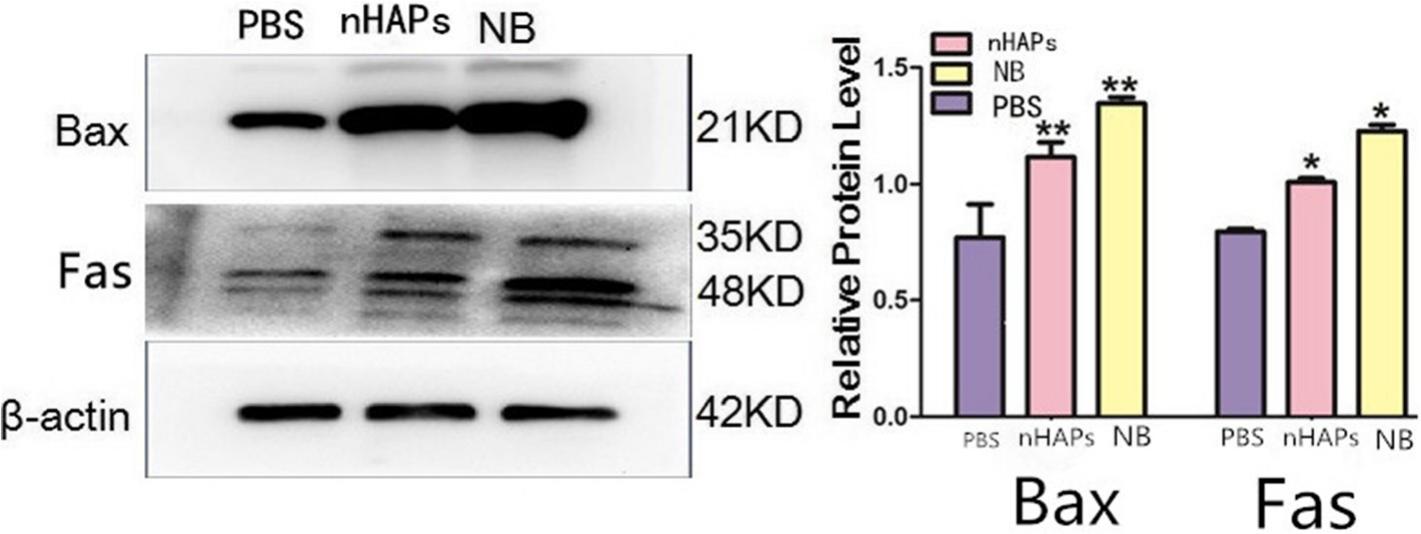
**Protein expression levels of Bax and Fas in JAR cells.** *p <0.05, **p <0.01.

Following the 48-h treatment, the expression of Bax and Fas proteins of JAR cells in the NB and nHAP groups was significantly higher than that in the control group (*p* <0.01 and *p* <0.05, respectively), and it was greater in the NB group than that in the nHAP group (*p* <0.01 and *p* <0.05, respectively) (Figure 5).

## Discussion

The previous results indicated that NB was related to placenta calcification obviously. And in this study, we found that cytotoxicity induced by NB was mediated via apoptosis and necrosis as verified by the presence of annexin V-positive and PI-positive cells and JAR cell ultrastructural changes (Figure 4). It is well known that the increase in Bax expression can promote cell apoptosis [17], and the mechanism of apoptosis is divided into membrane receptor pathway and mitochondrial pathway. By combining FasL and FasR, the former could cause caspase cascade activation to trigger apoptosis [18]. So, in this study, we chose FasR and Bax to explore the possible mechanism of JAR cell apoptosis. The results indicated that NB and nHAPs could cause JAR cells to significantly express the Bax protein (Figure 5). We also noticed that NB could cause the necrosis of the JAR cells (Figure 4e). The expression of Fas in the NB and nHAP groups was significantly higher than that in the control group (Figure 5). This is a potential indicator that NB can trigger apoptosis and necrosis in JAR cells through the binding of FasL to the FasR signaling pathway. So, we thought that NB, which has the biomineralization function, gathered the apoptotic body and the products of necrosis cells as the core, grew constantly, and caused the placenta calcification [19]. Furthermore, in the NB group, autophagy in JAR cells was apparent (Figure 4d), but this was not observed for the other groups. We are the first to report this particular phenomenon and we propose that NB possibly promote autophagy in JAR cells. The results showed that NB may be associated with the formation of autophagy body. Its function is double. One is to promote apoptosis and necrosis. The regulatory genes of cell apoptosis also participated in the regulation of autophagy body, such as the Bcl-2, caspase 9, caspase 3, and C-myc [20]. Autophagy can activate cell apoptosis, and if not removed in time, it would induce cell necrosis. The other is to regulate cell metabolism and suppress apoptosis [21]. In our study, which function is autophagy? Further studies are required to elucidate the precise mechanisms of autophagy and its relationship with JAR cell apoptosis and necrosis [22].

## Conclusions

We have described, for the first time, the cytotoxic effects of human JAR cells induced by NB and nHAPs *in vitro*. While both NB and nHAPs induced cytotoxicity in JAR cells, the effects exerted by NB were more potent. The mechanisms of NB cytotoxicity were probably activated by FasL binding to the FasR signaling pathway. We also noticed that autophagy of JAR cells occurred in the NB group, indicating that autophagy has some association with NB and possibly with apoptosis and necrosis.

## Competing interests

The authors declare that they have no competing interests.

## Authors’ contributions

MZ, DZ, and SL conceived and designed the experiments. MZ, GX, CF, and JY performed the experiments. MZ, SL, and JS analyzed the data. MZ, SL, JS, and DZ wrote the paper. All authors read and approved the final manuscript.
